# Prospective Evaluation of Intraorbital Soft Tissue Atrophy after Posttraumatic Bone Reconstruction: A Risk Factor for Enophthalmos

**DOI:** 10.3390/jpm12081210

**Published:** 2022-07-25

**Authors:** Cristian Dinu, Tiberiu Tamas, Gabriela Agrigoroaei, Sebastian Stoia, Horia Opris, Simion Bran, Gabriel Armencea, Avram Manea

**Affiliations:** Department of Maxillofacial Surgery and Implantology, Faculty of Dentistry, “Iuliu Hațieganu” University of Medicine and Pharmacy, 400012 Cluj-Napoca, Romania; dinu_christian@yahoo.com (C.D.); gabriela.agrigoroaei@yahoo.com (G.A.); stoia_sebi@yahoo.com (S.S.); horia.opris@gmail.com (H.O.); dr_brans@umfcluj.ro (S.B.); garmencea@yahoo.com (G.A.); avram.manea@umfcluj.ro (A.M.)

**Keywords:** soft tissue, orbital fracture, trauma, reconstruction, PSI, individualized, enophthalmos

## Abstract

Orbital fractures are a common finding in facial trauma, and serious complications may arise when orbital reconstruction is not performed properly. The virtual planning can be used to print stereolithographic models or to manufacture patient-specific titanium orbital implants (PSIs) through the process of selective laser melting. This method is currently considered the most accurate technique for orbital reconstruction. Even with the most accurate techniques of bone reconstruction, there are still situations where enophthalmos is present postoperatively, and it may be produced by intraorbital soft tissue atrophy. The aim of this paper was to evaluate the orbital soft tissue after posttraumatic reconstruction of the orbital walls’ fractures. Ten patients diagnosed and treated for unilateral orbital fractures were included in this prospective study. A postoperative CT scan of the head region with thin slices (0.6 mm) and soft and bone tissue windows was performed after at least 6 months. After data processing, the STL files were exported, and the bony volume, intraorbital fat tissue volume, and the muscular tissue volume were measured. The volumes of the reconstructed orbit tissues were compared with the volumes of the healthy orbit tissues for each patient. Our findings conclude that a higher or a lower grade of fat and muscular tissue loss is present in all cases of reconstructed orbital fractures. This can stand as a guide for primary or secondary soft tissue augmentation in orbital reconstruction.

## 1. Introduction

Orbital fractures are a common finding in facial trauma. An increased percent of patients with orbital fractures undergo surgical treatment, but there are still cases which are treated conservatively due to the different reasons [1,2,3,4]. Serious complications may arise when orbital reconstruction is not performed properly. These include enophthalmos, hypoglobus, diplopia, restricted motility, and muscle entrapment [4,5,6,7,8].

The anatomy of the orbital cavity is complex, and the reconstructive procedures in the orbital region are particularly difficult. The S-shaped orbital floor and the transition zone between the medial and the inferior wall can be challenging for reconstruction. The aim of reconstruction is to perfectly restore the orbit in all three dimensions in order to avoid esthetic and functional complications [9,10].

The first step to orbital surgery is an adequate exposure of the internal orbital walls. Selecting the appropriate surgical approach is mandatory [11]. The reconstruction of the bony walls can be performed with resorbable and non-resorbable materials. The non-resorbable materials are preferred due to their predictability and stable results. Titanium implants can be found as standard preformed orbital plates or individualized orbital implants. The standard plates are available in different sizes and can provide good results as long as they are placed in the correct position. The individualized implants use computer-aided design (CAD), which shares a virtual plan based on the patient’s 3D scan. All types of CAD-based individualized orbital implants require preoperative computerized planning. The scan will be transferred to the planning software, wherein the unaffected orbit can be mirrored to fit the affected side. From this point, physical biomodels of the virtually reconstructed orbit can provide 3D models. On these models, titanium meshes can be designed, bent, and sterilized preoperatively, together with the biomodel for intraoperative application [12,13,14,15].

The virtual planning can be used to manufacture patient-specific titanium orbital implants (PSIs) through the process of selective laser melting. This method is currently considered the most accurate technique for orbital reconstruction [16,17]. The PSI is not malleable and avoids human error during the pre-bending stage. The implant is designed with rounded edges so that the implant does not traumatize the soft tissue. Moreover, navigational target points can be designed on the surface of the implant. All of these lead to a very precise implant that can be very accurately placed [18].

Even with the most accurate techniques of bone reconstruction, there are still situations where enophthalmos is present after reconstruction, and it may be produced by intraorbital soft tissue atrophy. Orbital soft tissues, including extraocular muscles and intraorbital fat, are the most important components of orbital contents, accounting for approximately 50% of orbital cavity volume [19,20]. The change in volume of the soft tissue, following orbital trauma, may lead to diplopia, restricted ocular movement, and even impairment of visual function [21,22]. Precise assessment of intraorbital soft tissue is essential for diagnosis of intraorbital disorders and can affect the evaluation of treatment and planning for surgical intervention [23,24,25,26,27,28]. Computerized tomography (CT) and magnetic resonance imaging (MRI) are the main imaging methods to examine intraorbital soft tissues. Several studies have focused on segmenting the interested tissues of orbit by using semiautomatic or even automatic approaches based on computer image-processing techniques [29,30,31,32]. However, due to the complicated structure and the small volume of the orbit, as well as the unclear boundary of soft tissues in CT images, effective and precise segmentation of intraorbital fat and extraocular muscles remains a tough task [33].

The aim of this paper was to evaluate the orbital soft tissue after posttraumatic reconstruction of the orbital walls’ fractures.

## 2. Materials and Methods

After applying the exclusion criteria, 10 patients diagnosed with unilateral orbital fractures were included in this study. All 10 were treated in the same hospital, by the same surgeon, with the same surgical approach. In 3 cases, patient-specific implants (PSI) were used. For the rest of them, we used pre-bended titanium meshes on stereolithographic models which were obtained through the mirroring technique. All patients were evaluated after a minimum of 6 months after surgery. A very low degree of enophthalmos was present in all cases.

Several parameters were taken into consideration for the analysis, among which were the sex of the patients, the number of affected orbital walls, the bilateral orbital bony volume, the volume of the bilateral orbital fat tissue, and the volume of the bilateral orbital muscles.

**Figure 1:**
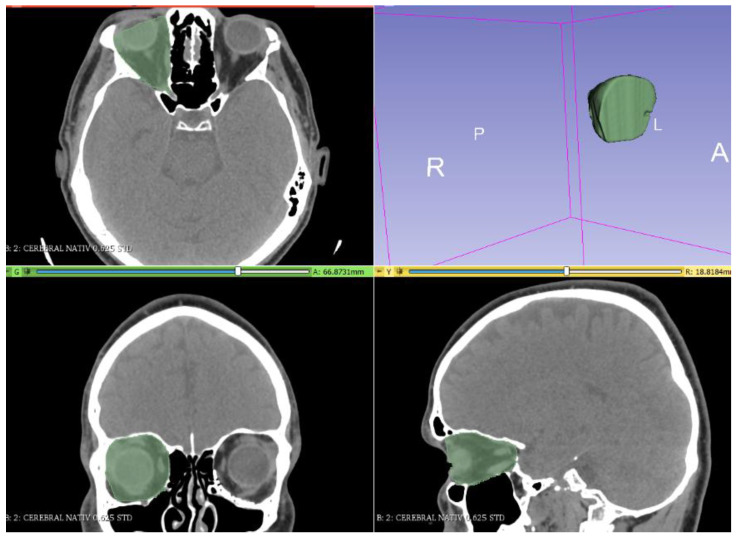
Segmentation of the volume of bony orbit.

**Figure 2:**
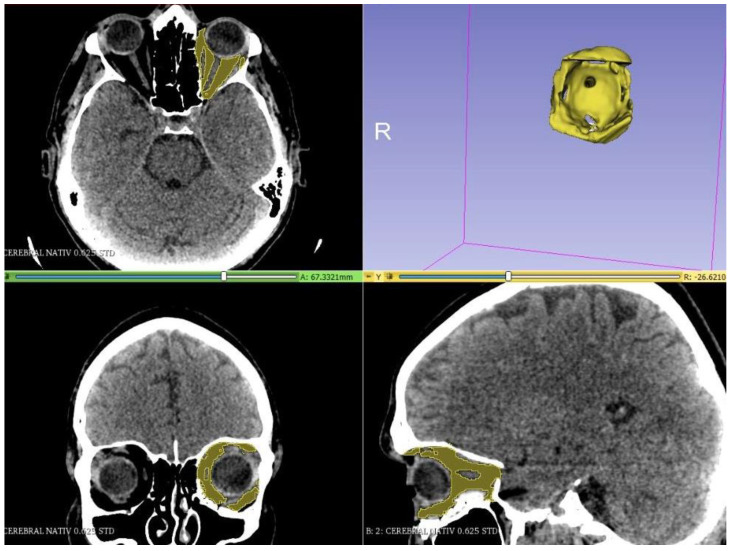
Segmentation of orbital fat tissue.

**Figure 3:**
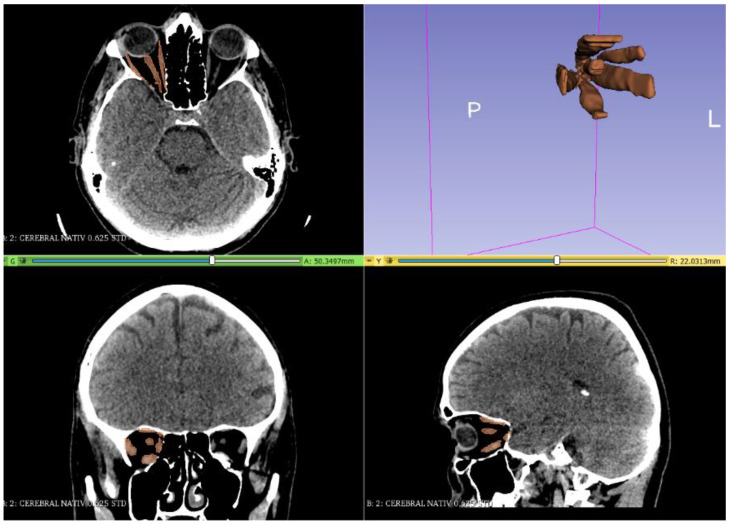
Segmentation of orbital muscles.

A CT scan of the head region with thin slices (0.6 mm), including soft and bone tissue windows, was performed. The DICOM (Digital Imaging and Communications in Medicine) files, resulting from the CT scan, were imported into the Slicer program, version 4.11, which is a semi-automatic software that is used to visualize and analyze the data and generate 3D segmentations. The densities used for segmentation (gray level) were as follows: from −200 to 100 Hounsfield units (HU) for the total orbital volume (Figure 1); −200 to 15 HU for the fat volume of the orbit (Figure 2); and −30 to 200 HU for muscle tissue volume (Figure 3).

**Figure 4:**
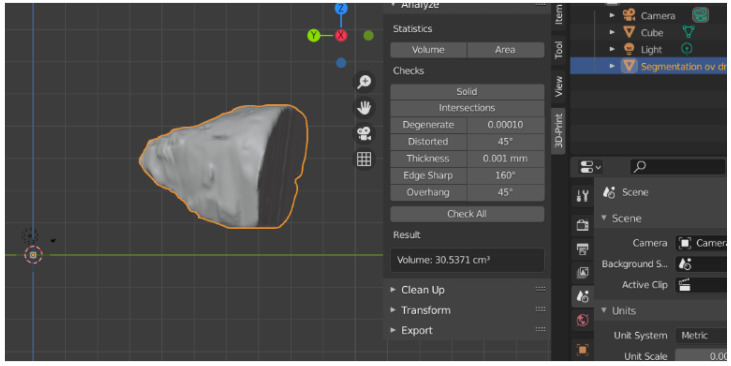
The volume of bony orbit in cm^3^.

**Figure 5:**
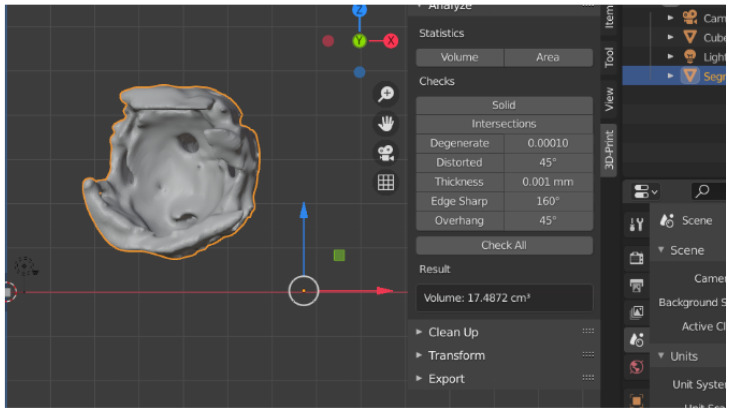
The volume of fat tissue in cm^3^.

**Figure 6:**
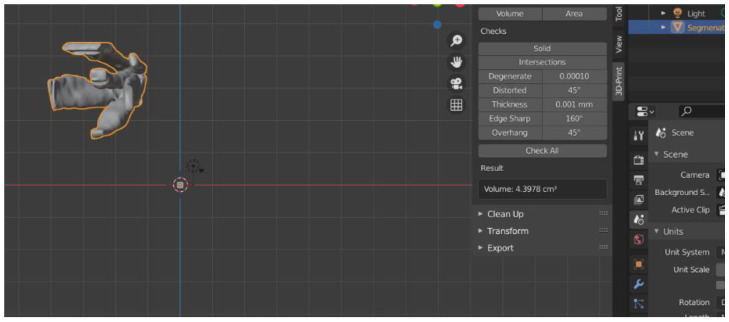
The volume of orbital muscles in cm^3^.

Afterward, the 3D reconstructions were exported as STL (Standard Triangle Language/Standard Tessellation Language) files, and the Blender 3D Software was used for volume calculation. The values obtained were measured in cubic centimeters (cm^3^) (Figure 4, Figure 5 and Figure 6).

## 3. Results

A total of 20 orbits were analyzed. The bony volume of the affected orbit (AOV), respectively of the healthy orbit (HOV), the fat volume of the affected orbit (AFV), respectively the fat volume of the healthy orbit (HFV), the muscles volume of the affected orbit (AMV), respectively the muscles volume of the healthy orbit (HMV), the number of fractured walls, and the sex of the patients are systematically presented, as follows in Table 1.

Regarding the number of fractured walls, 4 patients had one fractured wall, 4 patients had 2 fractured walls, and 2 patients had 3 fractured walls. We did not find any statistical correlation between the number of fractured walls and the volume of the soft tissue atrophy.

**Figure 7:**
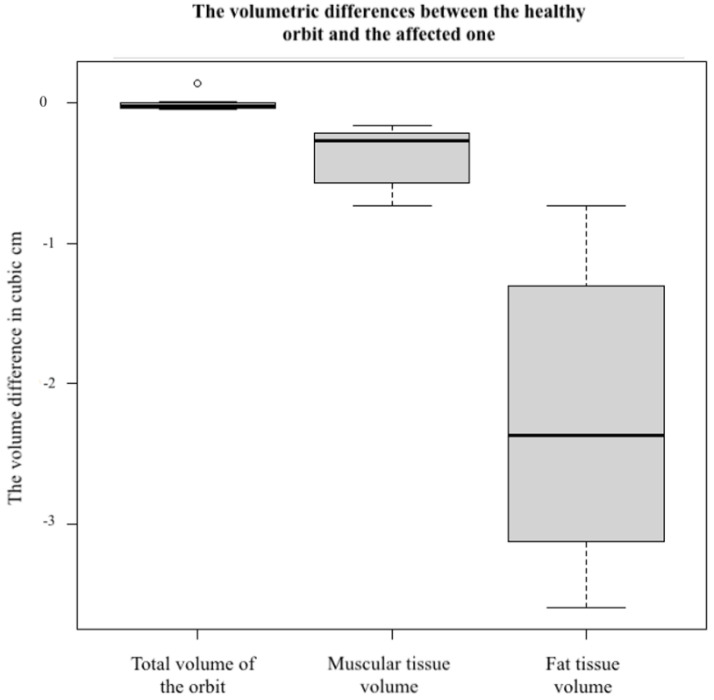
Volumetric differences between the healthy orbit and the affected one.

The differences (delta) between the affected orbit and the healthy orbit (control) are shown in Table 2, Table 3 and Table 4. Regarding the total orbital volume, the differences were minimal, with a maximum of 0.05 cm^3^ and a minimum of −0.14 cm^3^, without any correlation between a larger volume and the affected orbit. Referring to fat and muscular volume, it could be found that the volume of the affected orbit in the case of each variable was lower (delta with significant volumetric differences) compared to the healthy orbit (control) (Figure 7).

The volume of fat tissue was lower in all affected orbits, with a minimum of 0.73 cm^3^, maximum of 3.60 cm^3^, and mean of 2.228 cm^3^ (*p* < 0.001). Moreover, the musculature was affected in all fractured orbits. The minimum was 0.16 cm^3^, the maximum was 0.73 cm^3^, and the mean was 0.348 cm^3^ (*p* < 0.001).

Regarding the normal volume of the orbit, we found that females’ orbits were smaller, with a mean of 3.81 cm^3^; the fat volume was lower, with a mean of 3.75 cm^3^; and the muscle volume was also lower, with a mean of 0.68 cm^3^ (*p* < 0.01) (Table 5).

## 4. Discussion

The benefit of computer-assisted planning and computer-assisted surgery in orbital reconstruction has been well documented lately, permitting a safe procedure with predictable results. The orbit has a complex 3D shape. Even if we talk about printing stereolithographic models and pre-bending titanium meshes or printing directly a titanium implant, the results are much better than working without a CAD system [16,34,35].

Manually shaped titanium meshes achieve a very good approximation of the ideal reconstruction based on a 3D-printed model; however, designing and manufacturing the implants based on virtual models produces the most accurate implants [36,37,38]. Zimmerer et al. concluded that individualized orbital implants, particularly CAD-based ones, allow for a more precise posttraumatic orbital reconstruction than standard preformed implants [15]. We did not find a significant difference between these two, but our sample was smaller.

Poor primary reconstruction may lead to increased orbital volume and muscle restrictions. Persistent enophthalmos will lead to unacceptable aesthetic results. Diplopia is another functional poor outcome which is hardly accepted by the patient even if wearing correction glasses might slightly improve the situation. Although there is a debate whether these complications are mainly because of enlargement of the bony orbit or soft tissue atrophy, a good bony reconstruction and repositioning or augmentation of orbital soft tissues can improve clinical results [39,40].

There are also situations when the bony orbit is perfectly reconstructed, as we have shown, but the patient still presents enophthalmos due to the soft tissue atrophy. In these cases, estimating the need of overcorrection of the globe position depends mainly on the clinical evaluation. Our study found mean values for fat tissue and muscular tissue atrophy which can be useful for guiding the soft tissue correction.

For this correction, several autogenous and alloplastic materials can be used. The autogenous grafts can be time-consuming and involve donor site morbidity. The amount of volume reduction needed is difficult to estimate because the behavior of the soft tissue is different among patients [41].

Titanium spacers are designed in different sizes and represent a very good solution for compensation of orbital soft tissue atrophy. In their study, Spalthoff et al. concluded that augmentation of the intraorbital volume using titanium spacers remains a reasonable method to correct orbital deformities in delayed primary and secondary orbital reconstruction [42].

## 5. Conclusions

The anatomy of the orbital cavity is complex and needs very accurate reconstruction. Virtual planning and customized solutions, such as PSI or pre-bent titanium meshes conformed on stereolithographic models, offer predictable solutions and accurate results. Nevertheless, there are cases where very reduced enophthalmos is still present after surgical treatment. A higher or a lower grade of fat and muscular tissue loss is present in all cases of reconstructed orbital fractures. Our calculation of the mean value of lost volume can stand as a guide for primary or secondary soft tissue augmentation; however, larger studies are necessary.

## Figures and Tables

**Table 1:** Systematization of the main results.

Case No.	Affected Orbit	AOV	HOV	AFV	HFV	AMV	HMV	Number of Affected Walls	Gender
Case 1	R	27.15	27.01	13.91	14.64	3.48	4.21	1	F
Case 2	L	24.45	24.49	11.96	13.27	3.01	3.59	2	F
Case 3	L	25.15	25.14	7.93	10.16	3.79	3.95	1	F
Case 4	R	25.62	25.66	10.85	13.04	3.86	4.12	1	F
Case 5	L	30.52	30.54	14.36	17.49	4.20	4.39	2	M
Case 6	R	27.60	27.63	12.02	15.62	3.42	3.99	3	M
Case 7	R	28.03	28.02	14.42	15.19	4.40	4.67	3	M
Case 8	L	31.19	31.24	16.10	18.67	4.33	4.57	1	M
Case 9	R	30.47	30.46	13.41	16.68	4.43	4.70	2	M
Case 10	L	28.29	28.27	13.00	15.50	4.46	4.68	2	M

**Table 2:** Volume measurement of the bony orbit.

Case No.	HOV	AOV	Delta
Case 1	27.01	27.15	−0.14
Case 2	24.49	24.45	0.04
Case 3	25.14	25.15	−0.01
Case 4	25.66	25.62	0.04
Case 5	30.54	30.52	0.02
Case 6	27.63	27.60	0.03
Case 7	28.02	28.03	−0.01
Case 8	31.24	31.19	0.05
Case 9	30.46	30.47	−0.01
Case 10	28.29	28.27	0.02

**Table 3:** Volume measurement of the fat tissue.

Case No.	HFV	AFV	Delta
Case 1	14.64	13.91	0.73
Case 2	13.27	11.96	1.31
Case 3	10.16	7.93	2.23
Case 4	13.04	10.85	2.19
Case 5	17.49	14.36	3.12
Case 6	15.62	12.02	3.60
Case 7	15.19	14.42	0.77
Case 8	18.67	16.10	2.56
Case 9	16.68	13.41	3.27
Case 10	15.50	13.00	2.50

**Table 4:** Volume measurement of the muscular tissue.

Case No.	HMV	AMV	Delta
Case 1	4.21	3.48	0.73
Case 2	3.59	3.01	0.58
Case 3	3.95	3.79	0.16
Case 4	4.12	3.86	0.26
Case 5	4.39	4.20	0.19
Case 6	3.99	3.42	0.57
Case 7	4.67	4.40	0.27
Case 8	4.57	4.33	0.25
Case 9	4.70	4.43	0.27
Case 10	4.68	4.46	0.2

**Table 5:** Average and standard deviation.

Gender	HOV	AOV	HFV	AFV	HMV	HMV
F	25.57 ± 1.07	25.59 ± 1.14	12.78 ± 1.88	11.16 ± 2.50	3.97 ± 0.27	3.535 ± 0.39
M	29.38 ± 1.55	29.35 ± 1.55	16.53 ± 1.36	13.89 ± 1.41	4.50 ± 0.27	4.21 ± 0.4

## Data Availability

Not applicable.

## References

[1] Lemke B.N., Kikkawa D.O. (1999). Repair of orbital floor fractures with hydroxyapatite block scaffolding. Ophthal. Plast. Reconstr. Surg..

[2] Hwang K., You S.H., Sohn I.A. (2009). Analysis of orbital bone fractures: A 12-year study of 391 patients. J. Craniofac. Surg..

[3] Hwang K., Huan F., Hwang P.J. (2012). Diplopia and enophthalmos in blowout fractures. J. Craniofac. Surg..

[4] Ko M.J., Morris C.K., Kim J.W., Lad S.P., Arrigo R.T., Lad E.M. (2013). Orbital fractures: National inpatient trends and complications. Ophthalmic Plast. Reconstr. Surg..

[5] Biesman B.S., Hornblass A., Lisman R., Kazlas M. (1996). Diplopia after surgical repair of orbital floor fractures. Ophthalmic Plast. Reconstr. Surg..

[6] Hoşal B.M., Beatty R.L. (2002). Diplopia and enophthalmos after surgical repair of blowout fracture. Orbit.

[7] Chi M.J., Ku M., Shin K.H., Baek S. (2010). An analysis of 733 surgically treated blowout fractures. Ophthalmologica.

[8] Shin J.W., Lim J.S., Yoo G., Byeon J.H. (2013). An analysis of pure blowout fractures and associated ocular symptoms. J. Craniofacial Surg..

[9] Metzger M.C., Schön R., Zizelmann C., Weyer N., Gutwald R., Schmelzeisen R. (2007). Semiautomatic procedure for individual preforming of titanium meshes for orbital fractures. Plast. Reconstr. Surg..

[10] Baumann A., Sinko K., Dorner G. (2015). Late Reconstruction of the Orbit with Patient-Specific Implants Using Computer-Aided Planning and Navigation. J. Oral Maxillofac. Surg..

[11] Ellis E. (2014). Surgical approaches to the orbit in primary and secondary reconstruction. Facial Plast. Surg. FPS.

[12] Gellrich N.C., Schramm A., Hammer B., Rojas S., Cufi D., Lagrèze W., Schmelzeisen R. (2002). Computer-assisted secondary reconstruction of unilateral posttraumatic orbital deformity. Plast. Reconstr. Surg..

[13] Metzger M.C., Schön R., Weyer N., Rafii A., Gellrich N.C., Schmelzeisen R., Strong B.E. (2006). Anatomical 3-dimensional pre-bent titanium implant for orbital floor fractures. Ophthalmology.

[14] Zimmerer R.M., Gellrich N.C., von Bülow S., Strong E.B., Ellis E., Wagner M., Sanchez Aniceto G., Schramm A., Grant M.P., Thiam Chye L. (2018). Is there more to the clinical outcome in posttraumatic reconstruction of the inferior and medial orbital walls than accuracy of implant placement and implant surface contouring? A prospective multicenter study to identify predictors of clinical outcome. J. Cranio-Maxillo-Facial Surg..

[15] Zimmerer R.M., Ellis E., Aniceto G.S., Schramm A., Wagner M.E., Grant M.P., Cornelius C.P., Strong E.B., Rana M., Chye L.T. (2016). A prospective multicenter study to compare the precision of posttraumatic internal orbital reconstruction with standard preformed and individualized orbital implants. J. Cranio-Maxillo-Facial Surg..

[16] Rana M., Gellrich M.M., Gellrich N.C. (2015). Customised reconstruction of the orbital wall and engineering of selective laser melting (SLM) core implants. Br. J. Oral Maxillofac. Surg..

[17] Rana M., Chui C.H., Wagner M., Zimmerer R., Rana M., Gellrich N.C. (2015). Increasing the accuracy of orbital reconstruction with selective laser-melted patient-specific implants combined with intraoperative navigation. J. Oral Maxillofac. Surg..

[18] Strong E.B., Fuller S.C., Wiley D.F., Zumbansen J., Wilson M.D., Metzger M.C. (2013). Preformed vs. intraoperative bending of titanium mesh for orbital reconstruction. Otolaryngol.-Head Neck Surg..

[19] Du Y., Lu B.Y., Chen J., He J.F. (2019). Measurement of the Orbital Soft Tissue Volume in Chinese Adults Based on Three-Dimensional CT Reconstruction. J. Ophthalmol..

[20] Regensburg N.I., Wiersinga W.M., van Velthoven M.E., Berendschot T.T., Zonneveld F.W., Baldeschi L., Saeed P., Mourits M.P. (2011). Age and gender-specific reference values of orbital fat and muscle volumes in Caucasians. Br. J. Ophthalmol..

[21] Chazen J.L., Lantos J., Gupta A., Lelli G.J., Phillips C.D. (2014). Orbital soft-tissue trauma. Neuroimaging Clin. N. Am..

[22] Safi A.F., Richter M.T., Rothamel D., Nickenig H.J., Scheer M., Zöller J., Kreppel M. (2016). Influence of the volume of soft tissue herniation on clinical symptoms of patients with orbital floor fractures. J. Cranio-Maxillo-Facial Surg..

[23] Byun J.S., Moon N.J., Lee J.K. (2017). Quantitative analysis of orbital soft tissues on computed tomography to assess the activity of thyroid-associated orbitopathy. Graefe’s Arch. Clin. Exp. Ophthalmol..

[24] Bijlsma W.R., Mourits M.P. (2006). Radiologic measurement of extraocular muscle volumes in patients with Graves’ orbitopathy: A review and guideline. Orbit.

[25] Ye J., Kook K.H., Lee S.Y. (2006). Evaluation of computer-based volume measurement and porous polyethylene channel implants in reconstruction of large orbital wall fractures. Investig. Ophthalmol. Vis. Sci..

[26] Pilanci O., Ceran F., Sagir M., Teken A., Kuvat S.V. (2016). Evaluation of the Retro-Orbital Fatty Tissue Volume in Delayed Orbital Blow-Out Fractures. Ophthalmic Plast. Reconstr. Surg..

[27] Hu H., Xu X.Q., Liu H., Hong X.N., Shi H.B., Wu F.Y. (2017). Orbital benign and malignant lymphoproliferative disorders: Differentiation using semi-quantitative and quantitative analysis of dynamic contrast-enhanced magnetic resonance imaging. Eur. J. Radiol..

[28] Kim J.M., Chang M.H., Kyung S.E. (2015). The orbital volume measurement in patients with ventriculoperitoneal shunt. J. Craniofacial Surg..

[29] Regensburg N.I., Kok P.H., Zonneveld F.W., Baldeschi L., Saeed P., Wiersinga W.M., Mourits M.P. (2008). A new and validated CT-based method for the calculation of orbital soft tissue volumes. Investig. Ophthalmol. Vis. Sci..

[30] Bangiyev L., Raz E., Block T.K., Hagiwara M., Wu X., Yu E., Fatterpekar G.M. (2015). Evaluation of the orbit using contrast-enhanced radial 3D fat-suppressed T1 weighted gradient echo (Radial-VIBE) sequence. Br. J. Radiol..

[31] Comerci M., Elefante A., Strianese D., Senese R., Bonavolontà P., Alfano B., Bonavolontà B., Brunetti A. (2013). Semiautomatic regional segmentation to measure orbital fat volumes in thyroid-associated ophthalmopathy. A validation study. Neuroradiol. J..

[32] Jansen J., Schreurs R., Dubois L., Maal T.J., Gooris P.J., Becking A.G. (2016). Orbital volume analysis: Validation of a semi-automatic software segmentation method. Int. J. Comput. Assist. Radiol. Surg..

[33] Lutzemberger L., Salvetti O. (1998). Volumetric analysis of CT orbital images. Med. Biol. Eng. Comput..

[34] Rana M., Essig H., Rücker M., Gellrich N.C. (2012). Development and demonstration of a novel computer planning solution for predefined correction of enophthalmos in anophthalmic patients using prebended 3D titanium-meshes—A technical note. J. Oral Maxillofac. Surg..

[35] Mustafa S.F., Evans P.L., Bocca A., Patton D.W., Sugar A.W., Baxter P.W. (2011). Customized titanium reconstruction of post-traumatic orbital wall defects: A review of 22 cases. Int. J. Oral Maxillofac. Surg..

[36] Ramieri G., Spada M.C., Bianchi S.D., Berrone S. (2000). Dimensions and volumes of the orbit and orbital fat in posttraumatic enophthalmos. Dento Maxillo Facial Radiol..

[37] Manson P.N., Ruas E.J., Iliff N.T. (1987). Deep orbital reconstruction for correction of post-traumatic enophthalmos. Clin. Plast. Surg..

[38] Manson P.N., Grivas A., Rosenbaum A., Vannier M., Zinreich J., Iliff N. (1986). Studies on enophthalmos: II. The measurement of orbital injuries and their treatment by quantitative computed tomography. Plast. Reconstr. Surg..

[39] Brucoli M., Arcuri F., Cavenaghi R., Benech A. (2011). Analysis of complications after surgical repair of orbital fractures. J. Craniofacial Surg..

[40] Koo L., Hatton M.P., Rubin P.A. (2006). When is enophthalmos “significant”?. Ophthalmic Plast. Reconstr. Surg..

[41] Metzler P., Ezaldein H.H., Pfaff M.J., Parsaei Y., Steinbacher D.M. (2014). Correction of severe enophthalmos by simultaneous fat grafting and anatomic orbital reconstruction. J. Craniofacial Surg..

[42] Spalthoff S., Dittmann J., Zimmerer R., Jehn P., Tavassol F., Gellrich N.C. (2020). Intraorbital volume augmentation with patient-specific titanium spacers. J. Stomatol. Oral Maxillofac. Surg..

